# Th17 Cells in Autoimmune and Infectious Diseases

**DOI:** 10.1155/2014/651503

**Published:** 2014-08-03

**Authors:** José Francisco Zambrano-Zaragoza, Enrique Jhonatan Romo-Martínez, Ma. de Jesús Durán-Avelar, Noemí García-Magallanes, Norberto Vibanco-Pérez

**Affiliations:** ^1^Universidad Autónoma de Nayarit, Unidad Académica de Ciencias Químico Biológicas y Farmacéuticas, 63190 Tepic, NAY, Mexico; ^2^Universidad Politécnica de Sinaloa, Ingeniería en Biotecnología, 82199 Mazatlán, SIN, Mexico

## Abstract

The view of CD4 T-cell-mediated immunity as a balance between distinct lineages of Th1 and Th2 cells has changed dramatically. Identification of the IL-17 family of cytokines and of the fact that IL-23 mediates the expansion of IL-17-producing T cells uncovered a new subset of Th cells designated Th17 cells, which have emerged as a third independent T-cell subset that may play an essential role in protection against certain extracellular pathogens. Moreover, Th17 cells have been extensively analyzed because of their strong association with inflammatory disorders and autoimmune diseases. Also, they appear to be critical for controlling these disorders. Similar to Th1 and Th2 cells, Th17 cells require specific cytokines and transcription factors for their differentiation. Th17 cells have been characterized as one of the major pathogenic Th cell populations underlying the development of many autoimmune diseases, and they are enhanced and stabilized by IL-23. The characteristics of Th17 cells, cytokines, and their sources, as well as their role in infectious and autoimmune diseases, are discussed in this review.

## 1. Introduction

CD4+ T cells play an important role in the initiation of immune responses by providing help to other cells and taking on a variety of effector functions during immune reactions. Upon antigenic stimulation, naïve CD4+ T cells activate, expand, and differentiate into different effector subsets called T helpers—(Th) Th1, Th2, Th9, Th17, and Th22—that are characterized by the production of distinct cytokines and effector functions [1]. Th17 cells have been identified as one of the major pathogenic Th cell populations underlying the development of many autoimmune diseases, and it is known that IL-23 enhances and stabilizes them [2].

The main functions of the immune system are to recognize and subsequently eliminate foreign antigens, to induce immunologic memory, and to develop tolerance to self-antigens. Effective immunologic homeostasis relies on a continual balance among several factors, including Th cell activation and suppression by regulatory T cells (Treg). When homeostasis is disrupted and the immune system responds in favor of activation, the host becomes susceptible to autoimmunity [3].

The identification of the IL-17 family of cytokines and the finding that IL-23 mediates the expansion of IL-17-producing T cells led to the discovery of a new subset of Th cells designated Th17 cells. Similar to Th1 and Th2 cells, Th17 cells require specific cytokines and transcription factors for their differentiation.

Th17 cells have an important role in inducing the inflammatory process [3], the immediate protective response of the body to foreign pathogens; however, the immune response needs to be controlled to avoid injury mediated by the immune response in the form of chronic inflammation. CD4+ T cells are the first line of defense and they play a major role in the induction and regulation of immune responses, mainly by secreting cytokines. After antigenic stimulus, naïve CD4+ T cells may differentiate into effector T cells. Th1 and Th2 are the classical subsets involved in the immune response. Th1 secrete interferon-*γ* (IFN-*γ*) and interleukin (IL)-2, while Th2 produce IL-4, IL-13, and IL-5 [4, 5]. However, the T-cell subsets have been expanded, and Th17 cells have been described as a novel subset of the specialized Th cells lineage that produces IL-17 but not IFN-*γ* or IL-4 [6]. These cells are potent inducers of tissue inflammation and require TGF*β* in combination with other cytokines such as IL-6 and IL-23 for their differentiation [7].

## 2. Th17 Cells: Who Are They?

The T-cell subsets involved in inflammatory reactions are mainly Th1 and Th17. There is evidence that Th17 cells can be generated from effector memory CD4+ T cells. The involvement of such cytokines as IL-6, TGF*β*, IL-21, and IL-23 in the development of Th17 cells has been described clearly [8].

Th17 cells, first described in mice, are the major source of IL-17 in many types of adaptive immunity [6]. While Th1 and Th2 cells provide effector responses to intracellular bacterial infections and parasitic pathogens, respectively, Th17 cells offer protection against extracellular bacterial and fungal infections and have been implicated in autoimmunity. Th17 cells secrete different cytokines (IL-17A, IL-17F, IL-21, and IL-22) and their differentiation requires a novel set of transcription factors that includes a signal transducer and the activator of transcription 3 (Stat3), the retinoic acid receptor-related orphan receptor *γ* (ROR*γ*), the retinoic acid receptor-related orphan receptor a, the nuclear factor kappa-light-chain-enhancer of activated B (NF-kB) cells, a zeta inhibitor (IkBf), and basic leucine zipper transcription factor (Batf) [9, 10].

Th17 differentiation in mice requires initiation by TGF*β* and IL-6, expansion by IL-21, and stabilization by IL-23 [11]. In humans, the combination of TGF*β* and IL-21 was sufficient to induce differentiation from naïve T cells; indeed, TGF*β* plus IL-21 or TGF*β* plus IL-6 and IL-23 or IL-6 and IL-21 can induce expression of ROR*γ*. IL-1*β* plus IL-6 have been shown to be important in enhancing the amplification of Th17 cells and the production of IL-23 to maintain the Th17 cell population [4, 12].

### 2.1. The IL-17 Family

The IL-17 family comprises cytokines that participate in inflammatory responses and in the pathogenesis of many inflammatory disorders. There are six members in this family: IL-17A (also called IL-17 or CTLA8), IL-17B, IL-17C, IL-17D, IL-17E (or IL-25), and IL-17F. Their receptors form a family that contains five members (IL-17RA, IL-17RB, IL-17RC, IL-17RD, and IL-17RE). The IL-17 cytokines show high homology to IL-17A (16% to 50% of amino acid sequence identity), while the other members of this family and the IL-17 family receptors show structural homology among their members [5, 13].

IL-17A was discovered in 1993 and was found to have a homology to an open reading frame encoded within the Herpes virus Saimiri. IL-17A can lead to neutrophil recruitment, inflammation, and host defense, but pathological production leads to excessive inflammation and overt tissue damage [5, 14].

The cellular sources and regulation of IL-17F are similar to those of IL-17A. The genes that encode IL-17A and IL-17F are located on chromosome 6. IL-17E (or IL-25) shows the lowest similarity to IL-17A in terms of the amino acid sequence and also promotes Th2 cell-mediated immune responses, thereby contributing to allergic disease and defense against helminthic parasites [10, 15]. IL-17C is produced in epithelial cells and keratinocytes in response to pathogens or inflammatory cytokines and also promotes IL-17 production. Moreover, IL-17C induced TNF*α* and IL-1*β* production in the human monocytic cell line THP1 and mouse peritoneal exudate cells [16, 17]. In contrast, IL-17B and IL-17D are poorly studied and their biological functions are still unclear. However, forced expression of IL-17D in edited mouse tumor cells induced rejection by leading to the recruitment of NK cells [18].

### 2.2. Biological Functions of Members of the IL-17 Family

The IL-17 family's activities also include chronic inflammation associated with extracellular matrix destruction by activating the production of metalloproteinases and inhibiting extracellular matrix production in chondrocytes and osteoblasts. It has been reported that local mesenchymal cells promote the differentiation of naïve T cells into Th17 cells [19]. In inflammatory processes, IL-17 has shown synergistic interactions with other cytokines, such as TNF*α* and IL-1, leading to a chronic process [19].

The most thoroughly studied members of the IL-17 family are IL-17A and IL-17F; two molecules with similar biological activities that induce the production of proinflammatory cytokines, chemokines, antimicrobial peptides, and matrix metalloproteinases by activating innate and tissue resident cells, such as fibroblasts and epithelial cells. Additionally, IL-17A and IL-17F promote the recruitment and subsequent activation of neutrophils [20–22], and it has been observed that IL-17 sustains, rather than inducing, inflammation, thus amplifying the inflammatory response induced by a preexisting tissue injury [23]. On the other hand, IL-17A and IL-17F perform diverse immunoregulatory roles during infection by extracellular bacteria, fungi, and some types of viral infection [20, 21]. Interestingly, Maione et al. found evidence that IL-17A acts as a proaggregant agent by increasing platelet responses to ADP. They observed that IL-17A does not itselfcause an intra-arterial occlusive thrombus but could induce the endothelial features peculiar to a prothrombotic state, likely related to a downregulation of CD39 expression and activity in the vascular system [24, 25]. IL-17A also induces the expression of intercellular cell adhesion molecule 1 (ICAM-1) in keratinocytes and chondrocytes [21].

IL-17E (IL-25) produces a particularly important activity on acquired and innate immune responses not only because it is linked to allergic disease, but also because it plays a protective role in helminthic parasite infection. After antigen or pathogen stimulation, IL-17E induces production of Th2 cytokines such as IL-4, IL-5, and IL-13 by NKT, Th2, and Th9 cells. The role of IL-17B, IL-17C, and IL-17D in the immune system is still unclear, though they share a similar ability to induce inflammatory mediators. Both IL-17B and IL-17C induce TNF and IL-1b expression from a monocytic cell line and cause neutrophil infiltration. IL-17D induces expression of IL-6, IL-8, and GM-CSF in endothelial cells and inhibits hematopoietic progenitor colony formation [20, 21].

IL-17C is produced in epithelial cells and keratinocytes in response to pathogens or inflammatory cytokines and promotes IL-17 production. Moreover, IL-17C induced TNF*α* and IL-1*β* production in the human monocytic cell line THP1 and mouse peritoneal exudate cells [16, 17]. In contrast, IL-17B and IL-17D are poorly studied, so their biological functions remain unclear. However, forced expression of IL-17D in edited mouse tumor cells induced rejection by propitiating recruitment of NK cells [18].

The IL-17 family of cytokines mediates its biological functions via surface receptors on target cells. The IL-17R family contains 5 members that share sequence homology with IL-17RA. All members (IL-17RA, IL-17RB, IL-17RC, IL-17RD, and IL-17RE) have a fibronectin III-like domain in their extracellular part and an SEF/IL-17R (SEFIR) domain in their intracellular region. Functional receptors form heterodimers with IL-17RA as common subunit. IL-17RA is expressed constitutively in many cell types and is stimulated by IL-17 to induce production of proinflammatory molecules [13, 15].

In addition to Th17 cells, there are other immune cells that also produce IL-17, such as *γ* *δ* T cells [10, 19, 26], innate Th17 (iTh17) [27], natural killer (NK) cells, mast cells, and neutrophils [10, 19].

### 2.3. IL-17 Signaling

IL-17 upregulates the expression of proinflammatory chemokines and cytokines through activation of NF*κ*B, MAPKs, and the C/EBPs cascade. It also works with TNF*α* to induce gene expression and activates the JAK-PI3K and JAK-STAT pathways. In addition, IL-17A promotes inflammatory responses through the downregulation of microRNA-23b [5, 28]. In this way, although IL-17 does not initiate an inflammatory reaction while, if injected in preinflamed tissues, is able to further amplify biochemical and cellular events characteristic of the early stages of the inflammatory reaction [23].

Tumor-necrosis factor receptor-associated factor (TRAF6) is an E3 ubiquitin ligase essential for the activation of the NF*κ*B and MAPK pathways. Polyubiquitinated TRAF6 activates TGF*β*-activated kinase 1 (TAK1) with the subsequent NF*κ*B activation. However, IL-17RA does not contain a TRAF6 binding site, indicating the existence of another adaptor molecule that mediates the association of TRAF6 with IL-17RA [10, 15, 17]. At the C-terminus of the IL-17 receptor family there is a SEFIR domain. The STIR (SEFIR and TIR) domain superfamily includes TLRs, IL-1Rs, and IL-17 receptors. Interestingly, the SEFIR domain also interacts with a cytosolic protein called Act1 (NF*κ*B activator 1). Act1 is an NF*κ*B and IKK activator and an adapter for the recruitment of TRAF6. Indeed, Act1 is recruited to the IL-17 receptor complex through the homotypic interactions of the SEFIR domains upon IL-17 stimulation [13, 17]. Act1-deficient cells fail to activate NFkB and MAPKs upon IL-17A stimulation and thus cannot produce proinflammatory molecules, such as IL-6 and CXCL1. Since IL-17RA is required for IL-17F signaling, Act1 have a critical role in IL-17F signaling [10, 15].

Although the mechanism of activation of Act1 remains unclear, it is known that it mediates K63 ubiquitination and the activation of TRAF6. Moreover, IL-17A alone is a weak NF*κ*B activator but one that can synergize with other strong cytokines, such as TNF*α*, to promote and extend proinflammatory responses [5, 10].

Another component of the IL-17 signaling pathway is HSP90, which interacts with Act1 to mediate, as a scaffold protein, IL-17 signaling [5, 29]. Ubiquitin-specific processing protease 25 (USP25) is a negative regulator of the IL-17R signal transduction pathway because it restricts the ubiquitination status of TRAF6, thereby attenuating NFkB and MAPK signal transduction [13].

### 2.4. Cytokines Involved in Th17 Differentiation

#### 2.4.1. TGF*β*

TGF*β* (transforming growth factor-beta) is a pleiotropic factor with several different roles in T-cell development, homeostasis, and tolerance [30].

The role of TGF*β* in Th17 development and function has generated controversy. Recent studies support the existence of at least two functional subclasses of Th17 cells distinguished by their development in the presence or absence of TGF*β*, and there are reports that Th17 cells can produce their own TGF*β*, including TGF*β*1 and TGF*β*3, which would appear to exercise distinct programming functions [31].

The indispensability of TGF*β* in Th17 differentiation resurfaced later; this time in relation to the mouse, when it was reported that there may be two pathways of Th17 differentiation: a TGF*β*-dependent pathway that gives rise to “nonpathogenic” Th17 cells and a TGF*β*-independent pathway that gives rise to “pathogenic” Th17 cells [32]. Naïve precursors polarized in the presence of IL-6, IL-1*β*, and IL-23, but, in the absence of TGF*β* signaling, induced a population of so-called Th17 cells that induced EAE (experimental autoimmune encephalomyelitis) upon passive transfer into normal mice. In contrast, naïve cells polarized under identical conditions but with exogenous TGF*β*1 and no IL-23 (the so-called Th17(*β*) cells) and failed to induce EAE following transfers, despite expressing considerably higher amounts of IL-17A [33].

#### 2.4.2. IL-6

IL-6 is a pleiotropic cytokine secreted by the cells of the innate immune system such as DCs, monocytes, macrophages, mast cells, B cells, and a subset of activated T cells, though tumor cells, fibroblasts, endothelial cells, and keratinocytes also secrete IL-6 [7]. Recent studies have demonstrated that IL-6 has a very important role in regulating the balance between IL-17-producing Th17 cells and Treg. IL-6 (plus TGF*β*) induces the development of Th17 cells from naïve T cells; in contrast, IL-6 inhibits differentiation into Treg [34].

#### 2.4.3. IL-21

IL-21 is produced by a range of differentiated CD4+ T-cell subsets and natural killer (NK) T cells [35]. IL-21 signals through a heterodimeric receptor, which is formed by a common gamma chain (shared with IL-2, IL-4, IL-7, IL-9, IL-13, and IL-15 receptors) and an IL-21 specific receptor (IL-21R) [36, 37]. Since IL-21R is expressed on CD4+, CD8+ T cells, B cells, NK cells, dendritic cells, macrophages, and keratinocytes [36], it acts on a range of lymphoid lineages and exerts pleiotropic effects. IL-21 drives differentiation of naïve T cells into Th17 cells. IL-21 is induced by IL-6 and ROR*γ*t and stabilizes and maintains Th17 cells by upregulating its own expression and that of IL-23R [35, 38].

#### 2.4.4. IL-23

IL-23 is produced by activated dendritic cells and macrophages in response to microbial stimulation [39]. IL-23 appears to be the critical driver behind Th17 activation and the subsequent production of IL-17. IL-23 is a heterodimer of a unique IL-23p19 and shared IL-12/23p40 chains [40].

The signaling pathway of IL-23R has been described clearly. It involves Janus-associated kinase 2 (Jak2), tyrosine kinase 2 (Tyk2), and several members of the signal transducer activator of transcription (STAT) family, including STAT1, STAT3, STAT4, and STAT5 [41].

In lymphocytes, IL-23 induces a strong phosphorylation of STAT3 and a relatively weak activation of STAT4, whereas the reverse is true for IL-12-induced phosphorylation with respect to STAT4 and STAT3. Phosphorylation of STAT3 is essential for the development of IL-17-producing T-helper (Th17) cells, whereas STAT4 is important for increasing IFN*γ* production and the subsequent differentiation of Th1 cells [42].

## 3. Regulatory T Cells and Their Role in Th17 Cell Function

Regulatory T cells (Treg) are a subset of CD4+ lymphocytes involved in the maintenance of self-tolerance and the modulation of overall immune responses against infections and tumor cells by controlling CD4+ effector T cells. Treg secrete TGF*β* and IL-10 and require the specific cytokine TGF*β* and the transcription factor FoxP3 for their differentiation. While Th17 cells have been involved in the promotion of autoimmunity and Treg cells have been involved in the control of Th17 cells, the balance Th17/Treg has been judged important in the control of immunity mediated by Th17 cells [4]. Furthermore, both T-cell subsets require TGF*β*, Treg for the expression of FoxP3, and to induce the differentiation of Th17, in combination with IL-6 and IL-21. Consequently, in the proinflammatory environment (mediated by IL-6 or IL-21), ROR*γ*t expression is upregulated, while FoxP3 expression is reduced, and vice versa [34, 43].

On the other hand, Singh et al. have reported that aryl hydrocarbon receptor promotes epigenetic regulation thereby influencing reciprocal differentiation of Tregs and Th17 cells [44]; then it could be important in the maintenance of the Treg/Th17 ratios.

## 4. Th17 Cells in Autoimmune and Infectious Diseases

The role of Th17 cells in autoimmunity was demonstrated first in mice that were deficient for the p19 chain of the IL-23, in which the IL-17-producing T cells were significantly lower than in wild-type mice, highlighting the importance of the IL-23/Th17 axis in the pathogenicity of these autoimmune diseases [1]. Since then, the study of the pathogenic role of Th17 subset cells has focused on autoimmune inflammatory diseases, such as multiple sclerosis, rheumatoid arthritis, and psoriasis [45, 46]. The role of Th17 cells in different autoimmune, inflammatory, and infectious diseases is described below.

### 4.1. Glioma

Glioma is the most common malignant disease of the brain. Although the brain is believed to be immunologically privileged, increasing evidence shows that lymphocytes infiltrate the brain parenchyma during glioma formation and that the blood-brain barrier (BBB) is compromised under glioma stress. Few studies of the relationship between Th17 cells and this disease have been reported; however, research has shown that the numbers of Th17 cells appear to be higher than in control subjects. Moreover, Th17-related cytokines are expressed in glioma tissues, suggesting the role of these cells in glioma tumorigenesis and progression [47, 48]. Furthermore, the serum levels of IL-17 correlate with the disease, with age [49], and with the medium conditions of glioma cells that induce Th17 cell differentiation [47], thus supporting the role of Th17 cells in glioma.

### 4.2. Hashimoto's Thyroiditis

HT has long been epidemiologically associated with excessive iodine levels. However, the immunological mechanisms involved in this disease remain unclear. It has been reported that intrathyroid infiltrating Th17 cells and serum IL-17 levels increase significantly in HT patients. Moreover, the administration of moderately high levels of iodine was found to facilitate the polarization of murine splenic naïve T cells into Th17 cells, whereas extremely high levels of iodine favored Th1 polarization and inhibited Treg development, suggesting that both Th1 and Th17 cells may be involved in the pathogenesis of HT and that high levels of iodine may play a critical role in this process by modulating T-cell differentiation [50]. Additionally, IL-23 levels were found to be higher in patients with HT than controls [51, 52], while levels of IL-17A [50, 53, 54] and frequencies of Th17 cells were also higher in patients than controls [55, 56].

### 4.3. Atherosclerosis

Atherosclerosis is a chronic inflammatory disease regulated by T lymphocyte subsets. Th17 cells have been found to be elevated in patients [57, 58]. In addition, Th17-related cytokine correlates with the severity and progression of carotid artery plaques [58–61], and the Th17/Treg imbalance appears to be associated with plaque progression [62, 63]. Additionally, IL-17A has been involved in lipid metabolism and in the pathogenesis of atherosclerosis [64].

### 4.4. Multiple Sclerosis

MS is known as a neurotropic autoimmune disease in which a coordinated attack of innate and adaptive immune cells inflames the central nervous system (CNS) and interrupts signal transduction by demyelinating (destruction of the myelin sheath) the nerve fibers. This inflammatory demyelinating disease of the CNS has a certain autoimmune background [65]. T-helper cells play a critical role in disease onset and progression [66].

Several groups have studied and characterized T cells subsets and their cytokines in MS. They have reported that the frequency of Th17 [67, 68] and the levels of Th17 related cytokines [66, 69] were higher in MS patients compared to controls. Moreover, a lower Treg/Th17 ratio [65, 68] and a correlation of the severity of symptoms with the Treg/Th17 ratio [68] were also observed, suggesting their role in disease severity [65]. Additionally, it has been reported that the response of T cells to myelin antigen includes production of IL-17 [70]. Furthermore, the reduction of Th17 cells after treatment with IFN-*β* [66], methylprednisolone [68], anti-TNF therapy [71], fingolimod [72], and the suppression of the production of IL-23 by IFN-*β* treatment [73], together with the data described above, support the role of Th17 cells in this disease.

### 4.5. Type 1 Diabetes

DM1 is an autoimmune disease caused by T-cell-mediated destruction of insulin-producing cells. Although it has been thought that an imbalance between Th1 and Th2 is associated with the disease, the role of Th17 cells is under study [74]. As in MS, the Treg/Th17 balance has been found to be broken in DM1 patients; moreover the frequencies of TH17 cells seem to be higher in patients than controls [75].

It the case of* type 2 diabetes* (T2D), the alteration of the Th1/Th2/Th17/Treg paradigm may contribute to enhanced immune activation and inflammation and the subsequent development and progression of T2D [76]; moreover, glucoregulation may contribute to reducing IL-17 in patients [77].

### 4.6. Rheumatoid Arthritis

RA is a systemic autoimmune disease characterized by progressively destructive joint inflammation, destruction of articular cartilage, and bone and synovial hyperplasia. The chronic inflammation process is responsible for stimulating destructive mechanisms in the joint that causes structural damage and lead to functional disability and deterioration [78].

The contribution of Th17 cells to the development of chronic arthritis was first reported in mice. It was found that* in vivo* neutralization of IFN*γ* exacerbates Th17 induced arthritis, and anti-IL-17A treatment delays onset of arthritis induction by Th17 cells. Thus, Th17 cells may participate in the production of autoantibodies that can induce arthritis [79].

As in other autoimmune inflammatory diseases, TH17 frequencies were found to be increased in patients compared to controls [80, 81] as were the levels of IL-17 and IL-23 [81, 82]. Also, the notion that levels of Th17 cells could be reduced by anti-TNF [71], IL-21 [83], and IL-10 [84] has been reported.

### 4.7. Spondyloarthropathies

SpAs, now better known as spondyloarthritides are a diverse group of interrelated inflammatory arthritides. This group includes not only the prototypical disease, ankylosing spondylitis (AS), but also reactive arthritis, psoriatic arthritis, Chron's disease, undifferentiated SpA, and juvenile-onset spondyloarthritis [85]. The role of the IL-23/IL-17 axis in SpAs pathology has been reviewed extensively [86]; however, it has been reported that the serum levels of IL-17 and IL-23 were elevated in SpAs [87, 88]. Moreover, the circulating Th17 cells appear to be elevated as well [84, 87].

Another finding was that serum IL-17 and IL-23 levels in AS [89, 90] and the frequency of Th17 cells [91, 92] correlate with disease activity. As reported in other autoimmune diseases, response to treatment with anti-TNF therapy significantly reduces the frequency of TH17 cells [87].

### 4.8. Systemic Lupus Erythematosus

SLE is a systemic autoimmune disease of unknown etiology. There is increasing evidence that a disturbed T-cell homeostasis plays a critical role in the development of SLE. The main T-cell subsets that are pivotal for this T-cell balance consist of T-helper cells and regulatory T cells [93]. It has been suggested that an imbalance of circulating T-helper cells and an impairment of regulatory T cells are involved in the pathogenesis of SLE as has been reported for MS and DM1 [66, 75].

The role of Th17 cells in SLE has been supported by the higher serum levels of IL-17 [94, 95] and the higher frequency of circulating Th17 cells [95–97], although no differences between patients with the active and inactive forms of the disease has been found [93]. As has been reported for other diseases, the Treg/Th17 ratio was seen to be reduced in patients [96, 98].

Also, high levels of Th17 cytokines have been found in SLE patients [82]. Additionally, cytokine levels and Th17 frequencies correlate with disease activity [99, 100], and the imbalance between Treg and Th17 cells (Treg/Th17 ratio) correlates with disease activity as well [101, 102].

### 4.9. Psoriasis

Psoriasis is a chronic, relapsing, and immune-mediated inflammatory skin disease [2]. It is characterized by hyperplasia in the epidermis, infiltration of leukocytes, including monocytes, dendritic cells and T lymphocytes into both the dermis and the epidermis, and the dilation and growth of blood vessels [103]. Psoriasis is now defined as a Th1/Th17/Th22-based inflammatory disease [104]. The role of Th17 cells has been supported by the discovery of elevated frequencies of Th17 cells in patients and the fact that the Treg/Th17 ratio correlated with the skin lesions [103]. Moreover, IL-17A, the principal effector cytokine of Th17 cells, stimulates keratinocytes to produce chemokines, cytokines, and other proinflammatory mediators, thereby enabling IL-17A to bridge the innate and adaptive immune systems to sustain chronic inflammation [105]. Finally, this has been found to be elevated in patients with psoriasis [106].

Elevated frequencies of Th17 cells have been reported in psoriatic patients [103, 107]. As in other autoimmune diseases, the Treg/Th17 cells have been found to be deregulated, and this ratio correlates with disease activity [103]. Hence, clinical trials with IL-17 pathway inhibitors may provide a new therapeutic approach for patients with psoriasis [105, 108].

### 4.10. Vitiligo

Vitiligo is a common skin disorder, characterized by progressive skin depigmentation due to the loss of cutaneous melanocytes. The exact cause of melanocyte loss remains unclear, but a large number of observations have pointed to the important role of cellular immunity in vitiligo pathogenesis [109].

Th17 cells have been implicated in skin lesions in vitiligo [110] because of the discovery of higher levels of serum IL-17 in patients than controls [111, 112]. Th17 cell infiltration and decreased Tregs have also been reported [113]. Moreover, it has been found that levels of IL-17 decreased after treatment, while Foxp3 increased significantly [112], suggesting that the imbalance between Th17 and Treg could have an important role in vitiligo lesions.

### 4.11. Inflammatory Bowel Disease

Inflammatory bowel disease can be divided into two main forms: Crohn's disease (CD) and ulcerative colitis (UC). These are disabling diseases characterized by a chronic relapsing inflammatory response to commensal microflora in the gut [114, 115]. Although the mechanisms involved are still unclear, there is a clear genetic susceptibility [115]. In addition to the T-helper cell type (Th) 1 and Th2 immune responses, other subsets of T cells, namely, Th17 and regulatory T (Treg) cells, likely play a role in IBD, because the IL13/TH17 pathway has been postulated as an important biomarker of active IBD [17, 116], and the presence of IBD, but not the genetic load, alters mRNA expression of IBD-associated Th17/IL-13 genes [115]. Moreover, Th17 and Treg cells have been found in increased amounts in the peripheral blood of IBD patients [117], reaching levels that correlate with disease activity [118]. Also, the Treg/TH17 cell ratio was associated with disease activity in patients with Crohn's disease. Hence, together with the Treg/TH17 ratio, they could be considered as potential prognostic indicators [119].

### 4.12. Cardiovascular Diseases

The role of the IL-17 cytokine family in the pathogenesis of cardiovascular diseases has been described as one that amplifies both the inflammation induced by other cytokines in synergistic interactions [120] and the prothrombotic effects combined with the low FeCl3 concentrations that have been observed [25].

As in other pathologies, Th17 cells contribute to increasing cardiovasculopathies [121], while the Treg/Th17 imbalance has been associated with cardiovascular complications in uremic patients undergoing hemodialysis [122, 123].

### 4.13. Human Immunodeficiency Virus (HIV) Infection

The role of Th17 cells in the pathogenesis of HIV infection remains unclear. Selective depletion of this T-cell subset has been reported in gut-associated lymphoid tissue (GALT) as well as in the peripheral blood of HIV-infected individuals [124].

Th17 cells have been found to be associated with HIV patients in different ways. Studies have shown that Th17 cells are reduced in HIV patients [125, 126]. Additionally, the levels of Th17 cells appear to be higher in long-term nonprogressors compared to typical progressors [124]. Th17 cells and IL-17 levels have been shown to have a negative correlation with HIV plasma viral load [126, 127]. The Treg/Th17 ratio showed a negative correlation to viral plasma load [128, 129], although the percentage of Treg cells positively correlated with viral load before antiretroviral therapy [126]. Moreover, antiretroviral treatment normalizes the number of Th17 and the Treg/TH17 ratio in HIV patients [126, 130]. These data strongly suggest that Th17 cells and the Treg/Th17 balance could maintain HIV under control [131] and, therefore, could play a role in the pathogenesis of AIDS.

### 4.14. Hepatitis C Virus (HCV) Infection

The role of Th17 cells in HCV infection and progression remains unclear. It has been reported that Ag-specific Th17 cells are induced in patients infected by the hepatitis C virus (HCV) and that TGF*β* and IL-10, which are induced by the nonstructural viral protein 4 (NS4), suppressed Th17 responses in HCV-infected patients [132]. Moreover, higher levels of IL-17 have been found in patients compared to normal controls, although no correlation with the viremic state was found [133, 134].

Considering that IL-17 serum levels show correlations with serum alanine aminotransferase levels, an association of this cytokine with control of liver injury has been proposed [134], although Th17 cell expansion appears not to be associated with patients who were cured, who became persistently infected, or who had circulating levels of IL-17 in cases of fibrosis [135].

The effect of treatment with pegylated IFN plus ribavirin appears to be controversial, because of reports indicating that it does not affect IL-17 levels, and that there are no differences between responders and nonresponders [133]. Moreover, this treatment downmodulates the secretion of key Th1 and Th17 proinflammatory mediators and profibrotic growth factors as early as 12 weeks after treatment initiation [136].

### 4.15. Hepatitis B Virus (HBV) Infection

The role of Th17 cells in HBV infection has been documented by the expression of IL-23 and IL-23R in biopsied liver tissues from HBV-infected patients. Also, IL-17 appears to be indispensable for HBsAg-stimulated differentiation of naïve CD4(+) T cells into Th17 cells [137]. Thus, Th17 cells have been shown to participate in the pathogenesis of liver damage associated with the hepatitis B virus (HBV) [138].

The frequencies of Treg and Th17 cells are reported to increase in the peripheral blood of HBV patients [139, 140]. Th17 levels [141, 142] and the Treg/TH17 ratio appear to have a crucial role in the occurrence, development, and outcome of HBV [142, 143] and could be used as indicators of inflammation that may predict progression to fibrosis [144]. Hence, Th17 cells can contribute to immune activation and disease aggravation in patients with chronic HBV infection [138, 145], because of the correlation of Th17 cells with serum alanine aminotransferase levels [139]. However, this does not appear to occur in pediatric patients [140]. Additionally, Th17 cells and the IL-23/IL-17 axis seem to be involved in the acute or chronic form of the disease [146].

On the other hand, it was also found that IL-17A decreased the levels of HBVs antigen (HBsAg) and HBVe antigen (HBeAg) in culture medium, as well as the levels of intracellular HBV DNA in infected HepG2.2.15 cells [147], although treatment with telbivudine does not affect IL-17 levels [148]. In contrast, HBVc-Ag induces the production of IL-10, a cytokine involved in the blockage of Th17 cell activation [149]. Moreover, blockage of the IL-17 receptors (IL-17R) increased levels of HBsAg and extracellular HBV DNA in culture medium, as well as levels of intracellular HBV DNA [147].

The imbalance in the IL17/Il-13 axis has also been associated with responses to HBV vaccination in HCV-infected individuals [150].

## 5. Concluding Remarks

The role of Th17 cells in autoimmune diseases has been reported and supported with some clarity and has been shown to exhibit similar behaviors in the diseases studied. The number of diseases influenced by Th17 cells appears to be increasing. These diseases include those provoked by viral infections in which the role of Th17 cells remains unclear, though evidence suggests that they could play an important role in the control of these diseases.

## References

[1] Bettelli E, Korn T, Kuchroo VK (2007). Th17: the third member of the effector T cell trilogy. *Current Opinion in Immunology*.

[2] Duhen R, Glatigny S, Arbelaez CA, Blair TC, Oukka M, Bettelli E (2013). Cutting edge: the pathogenicity of IFN-*γ*-Producing Th17 cells is independent of T-bet. *Journal of Immunology*.

[3] Peck A, Mellins ED (2009). Breaking old paradigms: Th17 cells in autoimmune arthritis. *Clinical Immunology*.

[4] Noack M, Miossec P (2014). Th17 and regulatory T cell balance in autoimmune and inflammatory diseases. *Autoimmunity Reviews*.

[5] Song X, Qian Y (2013). The activation and regulation of IL-17 receptor mediated signaling. *Cytokine*.

[6] Park H, Li Z, Yang XO (2005). A distinct lineage of CD4 T cells regulates tissue inflammation by producing interleukin 17. *Nature Immunology*.

[7] Korn T, Bettelli E, Oukka M, Kuchroo VK (2009). *IL-17* and *Th17* cells. *Annual Review of Immunology*.

[8] Holzer U, Reinhardt K, Lang P, Handgretinger R, Fischer N (2013). Influence of a mutation in IFN-*γ* receptor 2 (IFNGR2) in human cells on the generation of Th17 cells in memory T cells. *Human Immunology*.

[9] Dong C (2011). Genetic controls of th17 cell differentiation and plasticity. *Experimental and Molecular Medicine*.

[10] Jin W, Dong C (2013). IL-17 cytokines in immunity and inflammation. *Emerging Microbes & Infections*.

[11] Hernández AS (2009). Helper (TH1, TH2, TH17) and regulatory cells (Treg, TH3, NKT) in rheumatoid arthritis. *Reumatologia Clinica Suplementos*.

[12] Yang L, Anderson DE, Baecher-Allan C (2008). IL-21 and TGF-*β* are required for differentiation of human T_H_17 cells. *Nature*.

[13] Gu C, Wu L, Li X (2013). IL-17 family: cytokines, receptors and signaling. *Cytokine*.

[14] Gaffen SL (2008). An overview of IL-17 function and signaling. *Cytokine*.

[15] Chang SH, Dong C (2011). Signaling of interleukin-17 family cytokines in immunity and inflammation. *Cellular Signalling*.

[16] Chang SH, Reynolds JM, Pappu BP, Chen G, Martinez GJ, Dong C (2011). Interleukin-17C promotes Th17 cell responses and autoimmune disease via interleukin-17 receptor E. *Immunity*.

[17] Song X, Qian Y (2013). IL-17 family cytokines mediated signaling in the pathogenesis of inflammatory diseases. *Cellular Signalling*.

[18] O'Sullivan T, Saddawi-Konefka R, Gross E (2014). Interleukin-17D mediates tumor rejection through recruitment of natural killer cells. *Cell Reports*.

[19] Miossec P, Kolls JK (2012). Targeting IL-17 and T H 17 cells in chronic inflammation. *Nature Reviews Drug Discovery*.

[20] Reynolds JM, Angkasekwinai P, Dong C (2010). IL-17 family member cytokines: regulation and function in innate immunity. *Cytokine and Growth Factor Reviews*.

[21] Iwakura Y, Ishigame H, Saijo S, Nakae S (2011). Functional specialization of interleukin-17 family members. *Immunity*.

[22] D'Acquisto F, Maione F, Pederzoli-Ribeil M (2010). From IL-15 to IL-33: the never-ending list of new players in inflammation. Is it time to forget the humble aspirin and move ahead?. *Biochemical Pharmacology*.

[23] Maione F, Paschalidis N, Mascolo N, Dufton N, Perretti M, D'Acquisto F (2009). Interleukin 17 sustains rather than induces inflammation. *Biochemical Pharmacology*.

[24] Maione F, Cicala C, Liverani E, Mascolo N, Perretti M, D'Acquisto F (2011). IL-17A increases ADP-induced platelet aggregation. *Biochemical and Biophysical Research Communications*.

[25] Maione F, Parisi A, Caiazzo E (2014). Interleukin-17A exacerbates ferric chloride-induced arterial thrombosis in rat carotid artery. *International Journal of Inflammation*.

[26] Sutton CE, Lalor SJ, Sweeney CM, Brereton CF, Lavelle EC, Mills KHG (2009). Interleukin-1 and IL-23 induce innate IL-17 production from *γδ* T cells, amplifying Th17 responses and autoimmunity. *Immunity*.

[27] Geddes K, Rubino SJ, Magalhaes JG (2011). Identification of an innate T helper type 17 response to intestinal bacterial pathogens. *Nature Medicine*.

[28] Zhu S, Pan W, Song X (2012). The microRNA miR-23b suppresses IL-17-associated autoimmune inflammation by targeting TAB2, TAB3 and IKK-*α*. *Nature Medicine*.

[29] Wang C, Wu L, Bulek K (2013). The psoriasis-associated D10N variant of the adaptor Act1 with impaired regulation by the molecular chaperone hsp90. *Nature Immunology*.

[30] Krstic J, Santibanez JF (2014). Transforming growth factor-beta and matrix metalloproteinases: functional interactions in tumor stroma-infiltrating myeloid cells. *The Scientific World Journal*.

[31] Ho AW, Gaffen SL (2010). IL-17RC: A partner in IL-17 signaling and beyond. *Seminars in Immunopathology*.

[32] Ghoreschi K, Laurence A, Yang X (2010). Generation of pathogenic TH17 cells in the absence of TGF-*β* signalling. *Nature*.

[33] Coombes JL, Siddiqui KRR, Arancibia-Cárcamo CV (2007). A functionally specialized population of mucosal CD103^+^ DCs induces Foxp3^+^ regulatory T cells via a TGF-*β* -and retinoic acid-dependent mechanism. *Journal of Experimental Medicine*.

[34] Bettelli E, Carrier Y, Gao W (2006). Reciprocal developmental pathways for the generation of pathogenic effector TH17 and regulatory T cells. *Nature*.

[35] Leonard WJ, Zeng R, Spolski R (2008). Interleukin 21: a cytokine/cytokine receptor system that has come of age. *Journal of Leukocyte Biology*.

[36] Spolski R, Leonard WJ (2008). The Yin and Yang of interleukin-21 in allergy, autoimmunity and cancer. *Current Opinion in Immunology*.

[37] Monteleone G, Pallone F, MacDonald TT (2008). Interleukin-21: a critical regulator of the balance between effector and regulatory T-cell responses. *Trends in Immunology*.

[38] Wu Z, Kim HP, Xue HH, Liu H, Zhao K, Leonard WJ (2005). Interleukin-21 receptor gene induction in human T cells is mediated by T-cell receptor-induced Sp1 activity. *Molecular and Cellular Biology*.

[39] Morrison PJ, Ballantyne SJ, Kullberg MC (2011). Interleukin-23 and T helper 17-type responses in intestinal inflammation: from cytokines to T-cell plasticity. *Immunology*.

[40] Lowes MA, Russell CB, Martin DA, Towne JE, Krueger JG (2013). The IL-23/T17 pathogenic axis in psoriasis is amplified by keratinocyte responses. *Trends in Immunology*.

[41] Parham C, Chirica M, Timans J (2002). A receptor for the heterodimeric cytokine IL-23 is composed of IL-12R*β*1 and a novel cytokine receptor subunit, IL-23R. *Journal of Immunology*.

[42] Yannam GR, Gutti T, Poluektova LY (2012). IL-23 in infections, inflammation, autoimmunity and cancer: possible role in HIV-1 and AIDS. *Journal of Neuroimmune Pharmacology*.

[43] Zhou L, Ivanov II, Spolski R (2007). IL-6 programs T_H_-17 cell differentiation by promoting sequential engagement of the IL-21 and IL-23 pathways. *Nature Immunology*.

[44] Singh NP, Singh UP, Singh B, Price RL, Nagarkatti M, Nagarkatti PS (2011). Activation of Aryl hydrocarbon receptor (AhR) leads to reciprocal epigenetic regulation of Foxp3 and IL-17 expression and amelioration of experimental colitis. *PLoS ONE*.

[45] Iwakura Y, Ishigame H (2006). The IL-23/IL-17 axis in inflammation. *Journal of Clinical Investigation*.

[46] Dardalhon V, Korn T, Kuchroo VK, Anderson AC (2008). Role of Th1 and Th17 cells in organ-specific autoimmunity. *Journal of Autoimmunity*.

[47] Paladugu M, Thakur A, Lum LG, Mittal S, Parajuli P (2013). Generation and immunologic functions of Th17 cells in malignant gliomas. *Cancer Immunology, Immunotherapy*.

[48] Cantini G, Pisati F, Mastropietro A (2011). A critical role for regulatory T cells in driving cytokine profiles of Th17 cells and their modulation of glioma microenvironment. *Cancer Immunology, Immunotherapy*.

[49] Doroudchi M, Pishe ZG, Malekzadeh M, Golmoghaddam H, Taghipour M, Ghaderi A (2013). Elevated serum IL-17A but not IL-6 in glioma versus meningioma and schwannoma. *Asian Pacific Journal of Cancer Prevention*.

[50] Li D, Cai W, Gu R (2013). Th17 cell plays a role in the pathogenesis of Hashimoto's thyroiditis in patients. *Clinical Immunology*.

[51] Ruggeri RM, Saitta S, Cristani M (2014). Serum interleukin-23 (IL-23) is increased in Hashimoto's thyroiditis. *Endocrine Journal*.

[52] Figueroa-Vega N, Alfonso-Pérez M, Benedicto I, Sánchez-Madrid F, González-Amaro R, Marazuela M (2010). Increased circulating pro-inflammatory cytokines and Th17 lymphocytes in Hashimoto's thyroiditis. *Journal of Clinical Endocrinology and Metabolism*.

[53] Guo H, Peng D, Yang XG (2014). A higher frequency of circulating IL-22^+^CD4^+^ T cells in Chinese patients with newly diagnosed Hashimoto's thyroiditis. *PLoS ONE*.

[54] Wang S, Baidoo SE, Liu Y (2013). T cell-derived leptin contributes to increased frequency of T helper type 17 cells in female patients with Hashimoto's thyroiditis. *Clinical and Experimental Immunology*.

[55] Bossowski A, Moniuszko M, Idźkowska E (2012). Evaluation of CD4^+^CD161^+^CD196^+^ and CD4^+^IL-17^+^ Th17 cells in the peripheral blood of young patients with Hashimoto’s thyroiditis and Grave’s disease. *Pediatric Endocrinology, Diabetes, and Metabolism*.

[56] Qin Q, Liu P, Liu L (2012). The increased but non-predominant expression of Th17- and Th1-specific cytokines in hashimoto's thyroiditis but not in graves' disease. *Brazilian Journal of Medical and Biological Research*.

[57] Zhang L, Wang T, Wang XQ (2013). Elevated frequencies of circulating Th22 cell in addition to Th17 cell and Th17/Th1 cell in patients with acute coronary syndrome. *PLoS ONE*.

[58] Newcomb DC, Boswell MG, Zhou W (2011). Human TH17 cells express a functional IL-13 receptor and IL-13 attenuates IL-17A production. *Journal of Allergy and Clinical Immunology*.

[59] Khojasteh-Fard M, Abolhalaj M, Amiri P (2012). IL-23 gene expression in PBMCs of patients with coronary artery disease. *Disease Markers*.

[60] Liu Z, Lu F, Pan H (2012). Correlation of peripheral Th17 cells and Th17-associated cytokines to the severity of carotid artery plaque and its clinical implication. *Atherosclerosis*.

[61] de Boer OJ, van der Meer JJ, Teeling P (2010). Differential expression of interleukin-17 family cytokines in intact and complicated human atherosclerotic plaques. *Journal of Pathology*.

[62] Liu Z, Zhao Y, Wei F (2014). Treatment with telmisartan/rosuvastatin combination has a beneficial synergistic effect on ameliorating Th17/Treg functional imbalance in hypertensive patients with carotid atherosclerosis. *Atherosclerosis*.

[63] Mengya Z, Hanyou M, Dong L, Xiaohong L, Lihua Z (2013). Th17/Treg imbalance induced by increased incidence of atherosclerosis in patients with Systemic Lupus Erythematosus (SLE). *Clinical Rheumatology*.

[64] Yu XH, Jiang N, Zheng XL, Cayabyab FS, Tang ZB, Tang CK (2014). Interleukin-17A in lipid metabolism and atherosclerosis. *Clinica Chimica Acta*.

[65] Jamshidian A, Shaygannejad V, Pourazar A, Zarkesh S, Gharagozloo M (2013). Biased Treg/Th17 balance away from regulatory toward inflammatory phenotype in relapsed multiple sclerosis and its correlation with severity of symptoms. *Journal of Neuroimmunology*.

[66] Esendagli G, Kurne AT, Sayat G, Kilic AK, Guc D, Karabudak R (2013). Evaluation of Th17-related cytokines and receptors in multiple sclerosis patients under interferon beta-1 therapy. *Journal of Neuroimmunology*.

[67] Brucklacher-Waldert V, Stuerner K, Kolster M, Wolthausen J, Tolosa E (2009). Phenotypical and functional characterization of T helper 17 cells in multiple sclerosis. *Brain*.

[68] Li Y, Wang H, Long Y, Lu Z, Hu X (2011). Increased memory Th17 cells in patients with neuromyelitis optica and multiple sclerosis. *Journal of Neuroimmunology*.

[69] Hedegaard CJ, Krakauer M, Bendtzen K, Lund H, Sellebjerg F, Nielsen CH (2008). T helper cell type 1 (Th1), Th2 and Th17 responses to myelin basic protein and disease activity in multiple sclerosis. *Immunology*.

[70] Vargas-Lowy D, Kivisäkk P, Gandhi R (2013). Increased Th17 response to myelin peptides in pediatric MS. *Clinical Immunology*.

[71] Aravena O, Pesce B, Soto L (2011). Anti-TNF therapy in patients with rheumatoid arthritis decreases Th1 and Th17 cell populations and expands IFN-*γ*-producing NK cell and regulatory T cell subsets. *Immunobiology*.

[72] Sato DK, Nakashima I, Bar-Or A (2014). Changes in Th17 and regulatory T cells after fingolimod initiation to treat multiple sclerosis. *Journal of Neuroimmunology*.

[73] Sweeney CM, Lonergan R, Basdeo SA (2011). IL-27 mediates the response to IFN-*β* therapy in multiple sclerosis patients by inhibiting Th17 cells. *Brain, Behavior, and Immunity*.

[74] Ryba-Stanisławowska M, Skrzypkowska M, MyŚliwiec M, MyŚliwska J (2013). Loss of the balance between CD4+Foxp3+ regulatory T cells and CD4+IL17A+ Th17 cells in patients with type 1 diabetes. *Human Immunology*.

[75] Ryba-Stanisławowska M, Skrzypkowska M, MyŚliwiec M, MyŚliwska J (2013). Loss of the balance between CD4^+^Foxp3^+^ regulatory T cells and CD4^+^IL17A^+^ Th17 cells in patients with type 1 diabetes. *Human Immunology*.

[76] Zhang C, Xiao C, Wang P (2014). The alteration of Th1/Th2/Th17/Treg paradigm in patients with type 2 diabetes mellitus: relationship with diabetic nephropathy. *Human Immunology*.

[77] Sumarac-Dumanovic M, Jeremic D, Pantovic A (2013). Therapeutic improvement of glucoregulation in newly diagnosed type 2 diabetes patients is associated with a reduction of IL-17 levels. *Immunobiology*.

[78] Paradowska-Gorycka A, Grzybowska-Kowalczyk A, Wojtecka-Lukasik E, Maslinski S (2010). IL-23 in the pathogenesis of rheumatoid arthritis. *Scandinavian Journal of Immunology*.

[79] Hickman-Brecks CL, Racz JL, Meyer DM, LaBranche TP, Allen PM (2011). Th17 cells can provide B cell help in autoantibody induced arthritis. *Journal of Autoimmunity*.

[80] Heo YJ, Joo YB, Oh HJ (2010). IL-10 suppresses Th17 cells and promotes regulatory T cells in the CD4+ T cell population of rheumatoid arthritis patients. *Immunology Letters*.

[81] Zizzo G, de Santis M, Bosello SL (2011). Synovial fluid-derived T helper 17 cells correlate with inflammatory activity in arthritis, irrespectively of diagnosis. *Clinical Immunology*.

[82] Estrada-Capetillo L, Hernandez-Castro B, Monsivais-Urenda A (2013). Induction of Th17 lymphocytes and Treg cells by monocyte-derived dendritic cells in patients with rheumatoid arthritis and systemic lupus erythematosus. *Clinical and Developmental Immunology*.

[83] Niu X, He D, Zhang X (2010). IL-21 regulates Th17 cells in rheumatoid arthritis. *Human Immunology*.

[84] Zhang L, Li Y-G, Qi L-H (2012). Increased frequencies of th22 cells as well as th17 cells in the peripheral blood of patients with ankylosing spondylitis and rheumatoid arthritis. *PLoS ONE*.

[85] Ehrenfeld M (2012). Spondyloarthropathies. *Best Practice and Research: Clinical Rheumatology*.

[86] Smith JA, Colbert RA (2014). Review: the interleukin-23/interleukin-17 axis in spondyloarthritis pathogenesis: Th17 and beyond. *Arthritis & Rheumatology*.

[87] Xueyi L, Lina C, Zhenbiao W, Qing H, Qiang L, Zhu P (2013). Levels of circulating Th17 cells and regulatory T cells in ankylosing spondylitis patients with an inadequate response to anti-TNF-*α* therapy. *Journal of Clinical Immunology*.

[88] Andersen T, Rasmussen TK, Hvid M (2012). Increased plasma levels of IL-21 and IL-23 in spondyloarthritis are not associated with clinical and MRI findings. *Rheumatology International*.

[89] Chen W, Chang Y, Lin K (2012). Association of serum interleukin-17 and interleukin-23 levels with disease activity in Chinese patients with ankylosing spondylitis. *Journal of the Chinese Medical Association*.

[90] Melis L, Vandooren B, Kruithof E (2010). Systemic levels of IL-23 are strongly associated with disease activity in rheumatoid arthritis but not spondyloarthritis. *Annals of the Rheumatic Diseases*.

[91] Bautista-Caro M, Arroyo-villa I, Castillo-gallego C (2013). Decreased Th17 and Th1 cells in the peripheral blood of patients with early non-radiographic axial spondyloarthritis: A marker of disease activity in HLA-B27+ patients. *Rheumatology*.

[92] Benham H, Norris P, Goodall J (2013). Th17 and Th22 cells in psoriatic arthritis and psoriasis. *Arthritis Research & Therapy*.

[93] Dolff S, Bijl M, Huitema MG, Limburg PC, Kallenberg CGM, Abdulahad WH (2011). Disturbed Th1, Th2, Th17 and Treg balance in patients with systemic lupus erythematosus. *Clinical Immunology*.

[94] Elewa EA, Zakaria O, Mohamed EI, Boghdadi G (2014). The role of interleukins 4, 17 and interferon gamma as biomarkers in patients with Systemic Lupus Erythematosus and their correlation with disease activity. *The Egyptian Rheumatologist*.

[95] Wong CK, Lit LCW, Tam LS, Li EKM, Wong PTY, Lam CWK (2008). Hyperproduction of IL-23 and IL-17 in patients with systemic lupus erythematosus: implications for Th17-mediated inflammation in auto-immunity. *Clinical Immunology*.

[96] Henriques A, Inês L, Couto M (2010). Frequency and functional activity of Th17, Tc17 and other T-cell subsets in Systemic Lupus Erythematosus. *Cellular Immunology*.

[97] Yang J, Chu Y, Yang X (2009). Th17 and natural treg cell population dynamics in systemic lupus erythematosus. *Arthritis and Rheumatism*.

[98] Vincze K, Kovats Z, Cseh A (2014). Peripheral CD4+ cell prevalence and pleuropulmonary manifestations in systemic lupus erythematosus patients. *Respiratory Medicine*.

[99] Yang X, Wang H, Zhao X, Wang L, Lv Q, Wang Q (2013). Th22, but not Th17 might be a good index to predict the tissue involvement of systemic lupus erythematosus. *Journal of Clinical Immunology*.

[100] Shah K, Lee W, Lee S (2010). Dysregulated balance of Th17 and Th1 cells in systemic lupus erythematosus. *Arthritis Research and Therapy*.

[101] Kleczynska W, Jakiela B, Plutecka H, Milewski M, Sanak M, Musial J (2011). Imbalance between Th17 and regulatory T-cells in systemic lupus erythematosus. *Folia Histochemica et Cytobiologica*.

[102] Wang B, Su H, Cui J, Li J (2012). Elevated Th17 cells are accompanied by FoxP3^+^ Treg cells decrease in patients with lupus nephritis. *Rheumatology International*.

[103] Zhang L, Yang X, Cheng J, Hui R, Gao T (2010). Increased Th17 cells are accompanied by FoxP3^+^ Treg cell accumulation and correlated with psoriasis disease severity. *Clinical Immunology*.

[104] Golden JB, McCormick TS, Ward NL (2013). IL-17 in psoriasis: implications for therapy and cardiovascular co-morbidities. *Cytokine*.

[105] Lynde CW, Poulin Y, Vender R, Bourcier M, Khalil S (2014). Interleukin 17A: toward a new understanding of psoriasis pathogenesis. *Journal of the American Academy of Dermatology*.

[106] Michalak-Stoma A, Bartosinska J, Kowal M, Juszkiewicz-Borowiec M, Gerkowicz A, Chodorowska G (2013). Serum levels of selected Th17 and Th22 cytokines in psoriatic patients. *Disease Markers*.

[107] Kagami S, Rizzo HL, Lee JJ, Koguchi Y, Blauvelt A (2010). Circulating Th17, Th22, and Th1 cells are increased in psoriasis. *Journal of Investigative Dermatology*.

[108] Settesoldi A, Coppola M, Rogai F, Annese V (2014). Ustekinumab: moving the target from psoriasis to Crohn's disease. *Expert Review of Gastroenterology and Hepatology*.

[109] Wang CQF, Cruz-Inigo AE, Fuentes-Duculan J (2011). Th17 cells and activated dendritic cells are increased in vitiligo lesions. *PLoS ONE*.

[110] Cerhan JR, Liu-Mares W, Fredericksen ZS (2008). Genetic variation in tumor necrosis factor and the nuclear factor-*κ*B canonical pathway and risk of non-Hodgkin's lymphoma. *Cancer Epidemiology Biomarkers and Prevention*.

[111] Elela MA, Hegazy RA, Fawzy MM, Rashed LA, Rasheed H (2013). Interleukin 17, interleukin 22 and FoxP3 expression in tissue and serum of non-segmental vitiligo: a case- controlled study on eighty-four patients. *European Journal of Dermatology*.

[112] Hegazy RA, Fawzy MM, Gawdat HI, Samir N, Rashed LA (2014). T helper 17 and Tregs: a novel proposed mechanism for NB-UVB in vitiligo. *Experimental Dermatology*.

[113] Ono S, Tanizaki H, Otsuka A (2014). Coexistent skin lesions of vitiligo and psoriasis vulgaris. Immunohistochemical analyses for IL-17A-producing cells and regulatory T cells. *Acta Dermato-Venereologica*.

[114] Siakavellas SI, Bamias G (2012). Role of the IL-23/IL-17 axis in Crohn’s disease. *Discovery Medicine*.

[115] Fransen K, van Sommeren S, Westra HJ (2014). Correlation of genetic risk and messenger RNA expression in a Th17/IL23 pathway analysis in inflammatory bowel disease. *Inflammatory Bowel Diseases*.

[116] Rafa H, Saoula H, Belkhelfa M (2013). IL-23/IL-17A axis correlates with the nitric oxide pathway in inflammatory bowel disease: Immunomodulatory effect of retinoic acid. *Journal of Interferon and Cytokine Research*.

[117] Ueno A, Jijon H, Chan R (2013). Increased prevalence of circulating novel IL-17 secreting Foxp3 expressing CD4+ T cells and defective suppressive function of circulating Foxp3+ regulatory cells support plasticity between Th17 and regulatory T cells in inflammatory bowel disease patients. *Inflammatory Bowel Diseases*.

[118] Olsen T, Rismo R, Cui G, Goll R, Christiansen I, Florholmen J (2011). TH1 and TH17 interactions in untreated inflamed mucosa of inflammatory bowel disease, and their potential to mediate the inflammation. *Cytokine*.

[119] Chao K, Zhang S, Yao J (2014). Imbalances of CD4+ T-cell subgroups in Crohn's disease and their relationship with disease activity and prognosis. *Journal of Gastroenterology and Hepatology*.

[120] Ding H, Yang J, Yang J, Ding J, Chen P, Zhu P (2012). Interleukin-17 contributes to cardiovascular diseases. *Molecular Biology Reports*.

[121] Zhao RX, Li WJ, Lu YR (2014). Increased peripheral proinflammatory T helper subsets contribute to cardiovascular complications in diabetic patients. *Mediators of Inflammation*.

[122] Danyan C, Xiaolong H, Song L, Hua G, Weixue T, Ke L (2013). The effects of rhBMP-2 and Treg/Th17 functional disequilibrium in uremic patients with cardiovascular complication after maintenance hemodialysis. *International Journal of Artificial Organs*.

[123] Zhang J, Hua G, Zhang X, Tong R, Du X, Li Z (2010). Regulatory T cells/T-helper cell 17 functional imbalance in uraemic patients on maintenance haemodialysis: a pivotal link between microinflammation and adverse cardiovascular events: original Article. *Nephrology*.

[124] Salgado M, Rallón NI, Rodés B, López M, Soriano V, Benito JM (2011). Long-term non-progressors display a greater number of Th17 cells than HIV-infected typical progressors. *Clinical Immunology*.

[125] Alvarez Y, Tuen M, Nàdas A, Hioe CE (2012). In vitro restoration of Th17 response during HIV infection with an antiretroviral drug and Th17 differentiation cytokines. *AIDS Research and Human Retroviruses*.

[126] He Y, Li J, Zheng Y (2012). A randomized case-control study of dynamic changes in peripheral blood Th17/Treg cell balance and interleukin-17 levels in highly active antiretroviral-treated HIV type 1/AIDS patients. *AIDS Research and Human Retroviruses*.

[127] Singh A, Vajpayee M, Ali SA, Mojumdar K, Chauhan NK, Singh R (2012). HIV-1 diseases progression associated with loss of Th17 cells in subtype “C” infection. *Cytokine*.

[128] Chevalier MF, Petitjean G, Dunyach-Rémy C (2013). The Th17/Treg ratio, IL-1RA and sCD14 levels in primary HIV infection predict the T-cell activation set point in the absence of systemic microbial translocation. *PLoS Pathogens*.

[129] Li D, Chen J, Jia M (2011). Loss of balance between T helper type 17 and regulatory T cells in chronic human immunodeficiency virus infection. *Clinical and Experimental Immunology*.

[130] Kim CJ, McKinnon LR, Kovacs C (2013). Mucosal Th17 cell function is altered during HIV infection and is an independent predictor of systemic immune activation. *The Journal of Immunology*.

[131] Brandt L, Benfield T, Mens H (2011). Low level of regulatory T cells and maintenance of balance between regulatory T cells and TH17 cells in HIV-1-infected elite controllers. *Journal of Acquired Immune Deficiency Syndromes*.

[132] Rowan AG, Fletcher JM, Ryan EJ (2008). Hepatitis C virus-specific Th17 cells are suppressed by virus-induced TGF-*β*. *Journal of Immunology*.

[133] Fathy A, Ahmed AS, Metwally L, Hassan A (2011). T helper type 1/T helper type 17-related cytokines in chronic hepatitis C patients before and after interferon and ribavirin therapy. *Medical Principles and Practice*.

[134] Sousa GM, Oliveira IS, Andrade LJO, Sousa-Atta MLB, Paraná R, Atta AM (2012). Serum levels of Th17 associated cytokines in chronic hepatitis C virus infection. *Cytokine*.

[135] Foster RG, Golden-Mason L, Rutebemberwa A, Rosen HR (2012). Interleukin (IL)-17/IL-22-producing T cells enriched within the liver of patients with chronic hepatitis C viral (HCV) infection. *Digestive Diseases and Sciences*.

[136] Jimenez-Sousa MA, Almansa R, de La Fuente C (2010). Increased Th1, Th17 and pro-fibrotic responses in hepatitis C-infected patients are down-regulated after 12 weeks of treatment with pegylated interferon plus ribavirin. *European Cytokine Network*.

[137] Wang Q, Zhou J, Zhang B (2013). Hepatitis B virus induces IL-23 production in antigen presenting cells and causes liver damage via the IL-23/IL-17 axis. *PLoS Pathogens*.

[138] Yang B, Wang Y, Zhao C (2013). Increased Th17 cells and interleukin-17 contribute to immune activation and disease aggravation in patients with chronic hepatitis B virus infection. *Immunology Letters*.

[139] Li J, Shi J, Ren W, Wu W, Chen Z (2014). Regulatory role of CD4(+)CD25(+)Foxp3(+) regulatory T cells on IL-17-secreting T cells in chronic hepatitis B patients. *Digestive Diseases and Sciences*.

[140] Zhu S, Zhang H, Dong Y (2013). The correlation between T helper type 17 cells and clinical characters in Chinese paediatric patients with chronic hepatitis B. *Clinical and Experimental Immunology*.

[141] Wang L, Meng Q, Zou Z (2012). Increased frequency of circulating Th17 cells in acute-on-chronic hepatitis B liver failure. *Digestive Diseases and Sciences*.

[142] Sun HQ, Zhang JY, Zhang H, Zou ZS, Wang FS, Jia JH (2012). Increased Th17 cells contribute to disease progression in patients with HBV-associated liver cirrhosis. *Journal of Viral Hepatitis*.

[143] Xue-Song L, Cheng-Zhong L, Ying Z, Mo-Bin W (2012). Changes of Treg and Th17 cells balance in the development of acute and chronic hepatitis B virus infection. *BMC Gastroenterology*.

[144] Li J, Qiu SJ, She W (2012). Significance of the balance between regulatory T (Treg) and T helper 17 (Th17) cells during hepatitis B virus related liver fibrosis. *PLoS ONE*.

[145] Zhang G, Xie D, Lin B (2013). Imbalance of interleukin-17-producing CD4 T cells/regulatory T cells axis occurs in remission stage of patients with hepatitis B virus-related acute-on-chronic liver failure. *Journal of Gastroenterology and Hepatology*.

[146] Niu YH, Yin DL, Liu HL (2013). Restoring the Treg cell to Th17 cell ratio may alleviate HBVrelated acute-on-chronic liver failure. *World Journal of Gastroenterology*.

[147] Wang B, Zhao X, Fan Y, Zhang J, Zhao J, Wang K (2013). IL-17A but not IL-22 suppresses the replication of hepatitis B virus mediated by over-expression of MxA and OAS mRNA in the HepG2.2.15 cell line. *Antiviral Research*.

[148] Kim HY, Jhun JY, Cho ML (2013). Interleukin-6 upregulates Th17 response via mTOR/STAT3 pathway in acute-on-chronic hepatitis B liver failure. *Journal of Gastroenterology*.

[149] Li J, Wu W, Peng G (2010). HBcAg induces interleukin-10 production, inhibiting HBcAg-specific Th17 responses in chronic hepatitis B patients. *Immunology and Cell Biology*.

[150] Wang JM, Ma CJ, Li GY (2013). Tim-3 alters the balance of IL-12/IL-23 and drives TH17 cells: role in hepatitis B vaccine failure during hepatitis C infection. *Vaccine*.

